# Functional and Transcriptional Induction of Aquaporin-1 Gene by Hypoxia; Analysis of Promoter and Role of Hif-1α

**DOI:** 10.1371/journal.pone.0028385

**Published:** 2011-12-07

**Authors:** Irene Abreu-Rodríguez, Rocío Sánchez Silva, Ana Paula Martins, Graça Soveral, Juan José Toledo-Aral, José López-Barneo, Miriam Echevarría

**Affiliations:** 1 Instituto de Biomedicina de Sevilla (IBiS), Hospital Universitario Virgen del Rocío, CSIC, Departamento de Fisiología Médica y Biofísica, Universidad de Sevilla, Seville, Spain; 2 Departamento de Química, REQUIMTE, FCT-UNL, Caparica, Portugal; 3 Faculdade de Farmácia, Universidade de Lisboa, Lisbon, Portugal; 4 Centro de Investigación Biomédica en Red sobre Enfermedades Neurodegenerativas (CIBERNED), Seville, Spain; National Institute of Environmental Health Sciences, United States of America

## Abstract

Aquaporin-1 (AQP1) is a water channel that is highly expressed in tissues with rapid O_2_ transport. It has been reported that this protein contributes to gas permeation (CO_2_, NO and O_2_) through the plasma membrane. We show that hypoxia increases Aqp1 mRNA and protein levels in tissues, namely mouse brain and lung, and in cultured cells, the 9L glioma cell line. Stopped-flow light-scattering experiments confirmed an increase in the water permeability of 9L cells exposed to hypoxia, supporting the view that hypoxic Aqp1 up-regulation has a functional role. To investigate the molecular mechanisms underlying this regulatory process, transcriptional regulation was studied by transient transfections of mouse endothelial cells with a 1297 bp 5′ proximal Aqp1 promoter-luciferase construct. Incubation in hypoxia produced a dose- and time-dependent induction of luciferase activity that was also obtained after treatments with hypoxia mimetics (DMOG and CoCl_2_) and by overexpressing stabilized mutated forms of HIF-1α. Single mutations or full deletions of the three putative HIF binding domains present in the Aqp1 promoter partially reduced its responsiveness to hypoxia, and transfection with Hif-1α siRNA decreased the *in vitro* hypoxia induction of Aqp1 mRNA and protein levels. Our results indicate that HIF-1α participates in the hypoxic induction of AQP1. However, we also demonstrate that the activation of Aqp1 promoter by hypoxia is complex and multifactorial and suggest that besides HIF-1α other transcription factors might contribute to this regulatory process. These data provide a conceptual framework to support future research on the involvement of AQP1 in a range of pathophysiological conditions, including edema, tumor growth, and respiratory diseases.

## Introduction

During the last decade new functions, locations, and regulatory pathways have been described for AQP1. Its canonical function as a specific channel for water transport across cell membranes has become wider and it has been suggested that it contributes to transmembrane gas permeation (CO_2_, NO, NH3 and O_2_) in several different cell types [1]–[4]. AQP1 is expressed in tissues with high rates of gas transport, such as microvessel endothelium, alveolar epithelium, and O_2_/CO_2_ sensing chemoreceptor glomus cells in the carotid body and neonatal adrenal chromaffin cells [5], [6]. Expression of AQP1 is now known to be associated with inflammatory and neoplastic processes and has been detected in response to mechanical injury in numerous cells and tissues, including astrocytes, pneumocytes, lymphocytes, and several types of tumor cell [7]–[12], in which its presence had not previously suspected. It is known that expression of several AQPs in the central nervous system (CNS) is altered by exposure to hypoxia and subsequent reoxygenation [13], and that inhibition of Aqp1 expression using siRNA significantly reduces hypoxia-inducible angiogenesis in cultures of human retinal vascular endothelial cells [14].

Chronic hypoxia induces the expression of numerous genes by activation of hypoxia-inducible transcription factors (HIF), mainly HIF-1α and HIF-2α [15], [16]. These transcription factors, stabilized by hypoxia, play an essential role in numerous adaptive or pathophysiological processes, such as erythropoiesis, glycolysis, angiogenesis, inflammation and tumor progression, among others [16], [17]. Participation of HIF-1α in the transcriptional regulation of some AQPs has recently been demonstrated [11], [14], [18], [19]. For instance, in the cerebellum of rats subjected to hypoxia, increments in the mRNA and protein levels of vascular endothelial growth factor (VEGF) and aquaporin-4 (AQP4) were found to be closely associated to an increase in HIF-1α expression. Increased expression of AQP4 enhances water permeability of blood vessels and contributes to explaining edema formation [20]. Consistent with this, a direct correlation was observed between expression of AQP4 and of VEGF and HIF-1α in glioma cells and peritumoral edematous tissue [21]. In an ischemic/hypoxic model, traumatic brain injury induces HIF-1α awhich, in turn, up-regulates expression of AQP4 and AQP9. Additionally, inhibition of HIF-1α by 2-methoxyestradiol reduced the up-regulated levels of both these AQPs [18].

We have previously reported that hypoxia induces Aqp1 mRNA expression [4], and it is also known that pathological situations presenting tissue hypoxia stimulate the transcriptional expression of AQP1 [4], [11], [14]. However, the molecular mechanism underlying these phenomena, in particular the direct participation of HIF-1α, has not yet been established. In this paper, we study the hypoxic regulation of the AQP1 gene and analyze in detail the role of the Aqp1 promoter in this process. We have also investigated the involvement of HIF-1α in Aqp1 modulation.

## Materials and Methods

### Ethics Statement

Animals were anesthetized by injection of 350 mg/kg chloral hydrate and then sacrificed following the animal care protocols approved by the local ethics Committee of Virgen del Rocio University Hospital. Such protocols were described in detailed in the grant proposal which led to the award of the funding used to perform the present study, and were approved on April 2008 by Dr Cisneros as president of the Ethics Committee of the Virgen del Rocío University Hospital (act of approval N° 4/2008). In all the experiments, animals were cared for in accordance with the European guidelines (Directive 86/609/EEC).

### Cell Line and Culture Conditions

Rat gliosarcoma cells (9L) were kindly provided by Dr M.J. Merrill [22]. These cells were cultured in Dulbecco's Modified Eagle Medium (DMEM; GIBCO-BRL) with 4.5 g/L glucose, 4 mM L-glutamine and pyruvate supplemented with 10% fetal calf serum (GIBCO-BRL) and maintained at 37°C and in 5% CO_2_. The endothelial murine cell line (EOMA cells, ATCC catalog no.: CRL-2586) derived from a mixed hemangioendothelioma was cultured at 37°C and 10% CO_2_ in DMEM with 4.5 g/L glucose, supplemented with 4 mM L-glutamine and 10% fetal bovine serum (BioWhittaker, Belgium). Primary cultures of pulmonary artery smooth muscle cells were obtained following previously described protocols [23].

### Plasmid Construction for Reporter Assays

The firefly luciferase reporter plasmid pXP2 (kindly provided by Dr J.A. Pintor-Toro [24]) was used to assess the promoter activity of the 5′-flanking region of the mouse AQP1 gene. An Aqp1 promoter construct −1297/−1 (P-AQP1) was generated by standard PCR from mouse genomic DNA using specific primers (S: 5′-GGTCTTTACTCGGGATTTTG-3′ and AS: 5′-GCTGGCAGGGACCTCGACTTA-3′) and first cloned into the pCR2.1 (Invitrogen, CA) vector, and then transferred by restriction with KpnI and XhoI into the pXP2 vector. The ∼1.3 Kb Aqp1 promoter-luciferase construct was digested by restriction enzymes to obtain smaller constructs. Restriction with SmaI, with SmaI/BbvCI and with SmaI/StuI produced the −650/−1, the −266/−1 and the −122/−1 constructs respectively. For nucleotide mutations of the three putative HIF binding sites (HBS) (CGTG) present on the ∼1.3 Kb promoter the QuickChange Site-Directed Mutagenesis Kit (Stratagene, CA) and the primers indicated in the Supplementary Table S1 were used. Mutations were confirmed by sequencing (Eurofins MWG, Ebersberg, Germany).

### Transient Transfection and Luminescence Assay

The day before transfection, EOMA cells were plated at 1.3×10^5^ cells/ml on 35-mm culture dishes, using DMEM culture medium without antibiotics and half the amount of serum. For transfection, 5 ml of Lipofectamine 2000 (Invitrogen) and 1 mg of the Aqp1 promoter-luciferase constructs were mixed in 500 ml of Opti-Mem (Invitrogen), together with 100 ng of pRL-TK vector (Promega, USA) to normalize for transfection efficiency. Transfection was allowed to proceed for 6 h and then the medium was replaced by complete DMEM. After overnight incubation, cells were left in normoxia or incubated in hypoxia prior to luciferase activity assays. When deleted constructs of the promoter were transfected, equimolar ratios of DNA with respect to the 1.3 Kb promoter were maintained using the pCDNA3 (Invitrogen) empty vector. In co-transfection experiments of Aqp1 promoter with mutated Hif-1α or Hif-2α DNA vectors (kindly provided by Dr C.W. Pugh and Dr C. Simon, respectively) [25], [26], 1 mg of each DNA was used. As control, 1 mg of the promoterless pXP2 vector was transfected or untransfected cells were used. The pH3SVL vector (kindly provided by Dr R. Wenger) [27] a modified pGL3b (Promega, WI) firefly luciferase plasmid with six Hif Binding Sites (HBS), was used as control for positive response to HIF (data not shown). Luciferase activity was measured with the GloMax™ 96 microplate luminometer (Promega) using the commercial Dual-Luciferase Reporter Assay System (Promega), according to manufacturer's instructions. Promoter luciferase activity was normalized against the renilla activity from pRL-TK vector.

### RNA Extraction and Reverse Transcription – Quantitative PCR (RT-qPCR) Analysis

TRIzol reagent (Invitrogen) was used following the manufacturer's protocol. The reverse transcription reaction was performed using the SuperScript II RNase H^−^ Reverse Transcriptase Kit (Invitrogen). Quantitative PCR analysis was performed in an ABI Prism 7500 Sequence Detection System (Applied Biosystems, Warrington, UK) using SYBR Green PCR Master Mix (Applied Biosystems) and the thermocycler conditions recommended by the manufacturer. Cyclophilin RNA was amplified to normalize for the amount of RNA used. Primers were designed using the Primer Express software and the corresponding sequences are indicated in the Supplementary Table S2. Melting curve analysis showed a single sharp peak with the expected Tm for all samples.

### Hypoxia Treatments

Experiments performed to measure Aqp1 promoter activity by Luciferase reporter assay and to evaluate stabilization of HIF-1α protein were carried out in a sealed anaerobic glove-box (COY Laboratory Products, MI) with O_2_ maintained at 1–2%. Mice were kept either under normal conditions or in a hypoxia chamber (COY Laboratory Products) at 10% O_2_ for 48 hours. Animals were anesthetized by injection of 350 mg/kg chloral hydrate and then sacrificed following the animal care protocols approved by our institution. Tissue samples were collected for later RNA extraction.

### In Situ Hybridization

Whole brains were dissected and fixed overnight at 4°C in 4% (wt/vol) paraformaldehyde in PBS. After fixing, brains were washed in PBS. In situ hybridization was performed on 30- to 50-µm thick slices cut with a Lancer vibratome as previously described [28]. Sense and antisense digoxigenin-UTP-labeled riboprobes against mouse *Aqp1* were synthesized from pGEM-T Easy vector containing a partial cDNA sequence (737 bp) of the gene, using SP6 or T7 RNApolymerase respectively. After hybridization, slices were incubated with alkaline phosphatase-conjugated anti-digoxigenin antibody (1∶1000; Roch*e*, Mannheim, Germany). Photographs were taken with a Provis microscope (Olympus, Tokyo, Japan) with Nomarski optics.

### Western blot analysis

Cells at ∼80% confluence were washed with cold PBS, scrapped and collected in 1 ml of cold PBS and centrifuged at 165× g for 5 min at 4°C. For protein extraction, each pellet was lysed in 150–300 µl of homogenization buffer as previously reported [4]. Proteins (10 mg) were resolved by SDS-PAGE (6%) for HIF-1α analysis and after electrophoresis were transferred onto Hybond-P PVDF membranes (Amersham Biosciences, NJ) using the Novex transfer system (Novel Experimental Technology, CA). Membranes were probed overnight with 1∶500 anti-HIF-1α (Cayman Chemical, MI) and 1∶5000 anti-β actin (Abcam, UK) as primary antibodies and after washing they were incubated for 2 hours with the corresponding anti-IgG horseradish peroxidase secondary antibodies. The immunoreactive bands were developed with the ECL PLUS Western Blot Detection System (GE Healthcare, USA) and visualized using a Typhoon 9400 phosphorimager (Amersham Biosciences).

### Immunocytochemistry

Cells were plated on poly L-lysine coated coverslips. After incubation in hypoxic or normoxic conditions, they were washed with PBS and fixed for 10 min with 3% paraformaldehyde at room temperature. Cells were then permeabilized with triton 0.1% in PBS (PBTx) for 5 min and blocked for 2 hours with 10% fetal calf serum and 1 mg/ml BSA in PBTx before incubation overnight at 4°C with the primary antibody. Rabbit polyclonal anti-AQP1 at 1∶500 dilution (Abcam) was used, followed by the two-step EnVision+Dual Link System-HRP (DakoCytomation, Denmark), a staining technique using goat anti-rabbit immunoglobulins conjugated to a peroxidase-labeled polymer and DAB substrate/chromogen solution, to produce a brown precipitate. Samples were counter-stained with hematoxylin and slides were mounted in 50% glycerol in PBS with 0.02% sodium azide in mounting medium (DakoCytomation). Sections were photographed using an AX70-Olympus microscope equipped with an Olympus DP10 camera.

### Water Permeability Measurements

Osmotic water permeability (*P_f_*) was measured in suspensions of freshly suspended cells with or without hypoxia treatment, by stopped-flow light-scattering spectroscopy. Stopped-flow experiments were performed on a temperature-controlled Hi-Tech Scientific PQ/SF-53 spectrometer system (TgK Scientific, UK), which has a 2-ms dead time, and is interfaced with an 80386 IBM PC/AT-compatible microcomputer. Experiments were performed at temperatures ranging from 10°C to 32°C. Typically, five runs were stored and analyzed in each experimental condition. After the hypoxia or normoxia treatment, cells were detached from the flasks by incubation in PBS containing 20 mM EDTA for 15 min. Cells were then washed twice with PBS and suspended at a density of 1.5×10^3^ to 3.5×10^3^ cells/mm^3^. Cell suspensions were mixed with an equal amount of hyperosmotic solution (PBS containing 300 mM mannitol) to reach an inwardly directed gradient (150 mOsM) of the impermeant solute. Cell shrinkage due to water efflux causes an increase in scattered light intensity. The kinetics of cell shrinkage were assessed in terms of variation in the intensity of the light at 400 nm scattered at 90° [29] until the light signal stabilized. The *P_f_* was estimated by fitting the scattered light signal with a double exponential function and using the linear relationship between *P_f_* and the weighted average rate constant k_de_ obtained from the expression k_de_ = (ΔI_1_k_1_+ΔI_2_k_2_)/ΔI_1_+ΔI_2_), where ΔI_1_ and ΔI_2_ correspond to the changes in signal with a slow or a fast rate constant, k_1_ or k_2_, respectively. *P_f_* (cm s^−1^) was calculated from the equation *P_f_* = k_de_ (V_o_/A)(1/V_w_(osm_out_)_∞_) where *V_o_/A* is the cell volume to area ratio, *V_w_* is the molar volume of water and *(osm_out_)_∞_* is the final medium osmolarity after the applied osmotic gradient. All solution osmolarities were determined from freezing point depression on a semi-micro osmometer (Knauer, Germany). Standards of 100 and 400 mOsM were analyzed prior to samples, which were measured in triplicate.

### Activation Energy Evaluation

Water permeability was measured at five different temperatures ranging from 10°C to 32°C. The activation energy (E_a_) of water transport was calculated from the slope of the Arrhenius plot (lnP_f_ as a function of 1/T) multiplied by the gas constant R.

### Cell Volume Measurements

Equilibrium cell volumes were obtained by phase contrast microscopy using an inverted microscope (Axiovert Zeiss 100 M) equipped with a digital camera. Cells were dislodged with PBS-EDTA solution, washed and resuspended in PBS. For each set of data measured, an aliquot of cell suspension was placed on a microscope slide and an average of 6 images was taken with 4–6 cells in each. Cells were assumed to have a spherical shape with a diameter calculated as the average of the maximum and minimum dimensions of each cell.

### Statistical analysis

Data were presented as mean ± standard error of the mean (SEM), and were analyzed with either the paired Student's t-test or one-way analysis of variance (ANOVA) followed by Tukey's, Student-Newman-Keuls or Fisher's tests. All statistical calculations were performed using SigmaStat 2.03 for Windows. P values considered significant are marked with asterisks: P≤0.05 with one asterisk (*), P≤0.01 with two asterisks (**) and P≤0.001 with three asterisks (***).

## Results

### Molecular and Functional Induction of AQP1 by Hypoxia

**Figure 1:**
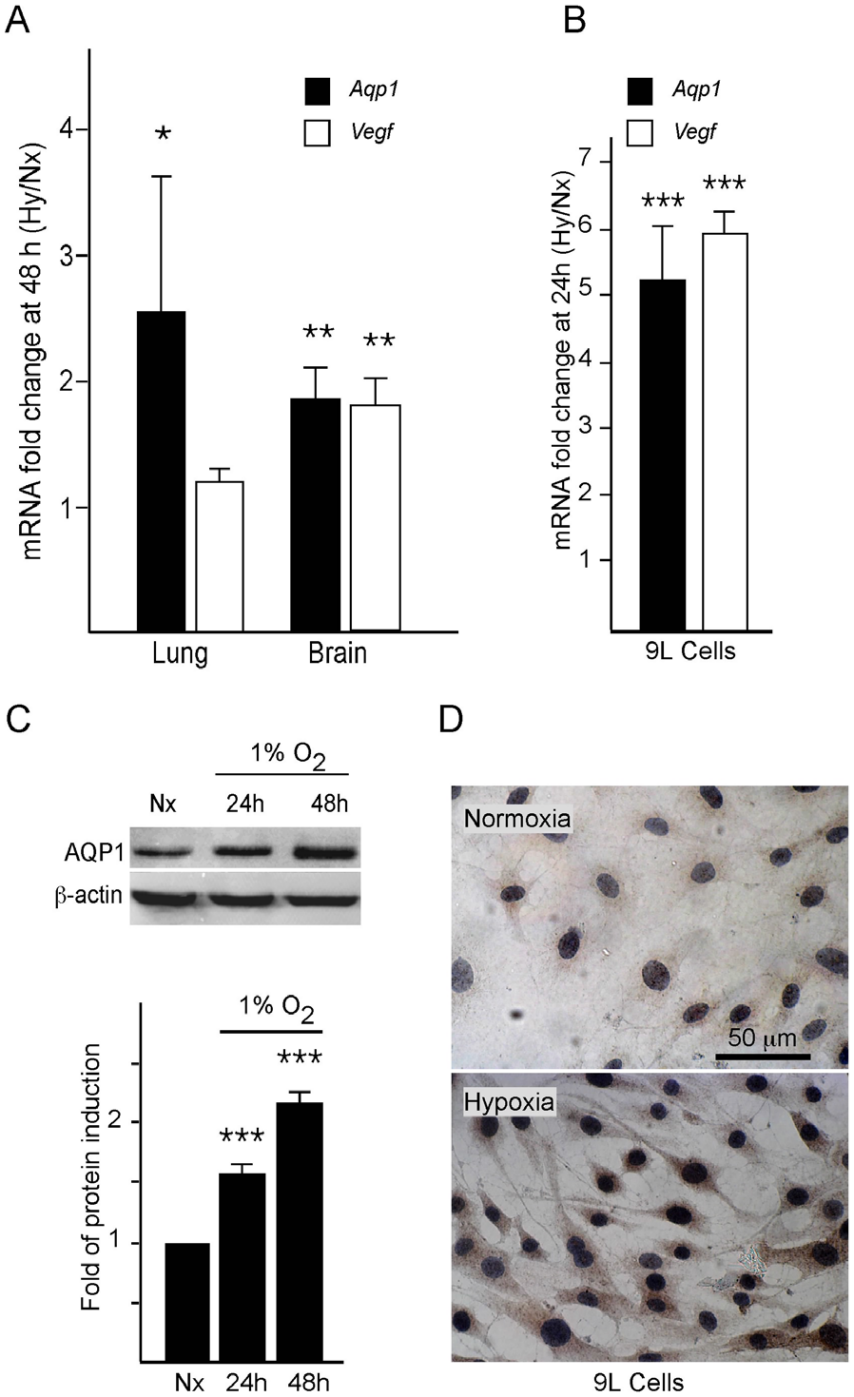
Hypoxic up-regulation of Aqp1 *in vivo*. (A) RT-qPCR analysis showing induction of Aqp1 and Vegf mRNA in lung and brain of mice exposed to hypoxia (10% O_2_ for 48 h, Hy) with respect to normoxia (48 h at 20% O_2_, Nx). (B) RT-qPCR analysis showing induction of Aqp1 and Vegf mRNAs in the glioblastoma (9L) cell line incubated in hypoxic (1% O_2_ for 24 h) or normoxic conditions. All values were normalized to the normoxic levels of mRNA and are presented as means ± SEM (N≥3), *P≤0.05, **P≤0.01, *** P≤0.001. (C) Representative western blot analysis of AQP1 in normoxic cells or after 24 and 48 h of hypoxic treatment (1% O_2_) and a summary of quantification data obtained from at least 3 independent experiments (N = 3). β-actin was used as control for protein loading. (D) Immunohistochemistry assay of AQP1 expression in 9L cells kept under normoxic conditions or exposed to hypoxia (1% O_2_ for 48 h). Brown immunostaining indicates the presence of AQP1; nuclei were stained with hematoxylin.

We have previously shown that Aqp1-mRNA expression in rat lungs is induced by the exposure of animals to hypoxia (10% O_2_) for 24 hours [4]. To determine whether this induction by hypoxia was a general phenomenon, similar experiments were performed in mice and in cultured cell lines. The RT-qPCR analysis confirmed that expression of Aqp1 increased by about 2.6- and 2.0-fold in mouse lung and brain respectively, after exposure to mild hypoxia (10% O_2_) for 48 hours (Figure 1A). Induction of vascular endothelial growth factor (Vegf), a well-established hypoxia-responsive gene, was analyzed as control. Although no induction of this gene was detected in lung tissue, it was observed that Vegf was induced faster than Aqp1 in brain tissue, indicating that expression kinetics of the two genes are different in this organ (Supplementary Figure S1A). Expression of Aqp1 in the lung mainly occurs in endothelium and vascular smooth muscle. In primary cultures of isolated rat pulmonary arterial myocytes hypoxia caused Aqp1 and VEGF to increase by ∼1.5 and 2.5-fold, respectively (Supplementary Figure S1B). In situ hybridization analysis showed that within the brain up-regulation of Aqp1 RNA was clearly detected in epithelial cells of the choroid plexus (Supplementary Figure S1C).

To gain further insight into the consequences of Aqp1 induction by hypoxia, we performed experiments in 9L cells, a glioblastoma cell line in which up-regulation of Aqp1 mRNA by hypoxia has also been reported [22]. Exposure of 9L cells to hypoxia (1% O_2_) for 24 to 48 hours resulted in a marked up-regulation of Aqp1 expression at mRNA and protein levels. Analysis by RT-qPCR showed more than a 5-fold induction of Aqp1 mRNA after 24 hours of hypoxic treatment, and, as expected, up-regulation of Vegf (∼6-fold) was also observed (Figure 1B). Importantly, higher expression of Aqp1 protein following hypoxia was also confirmed by western blot analysis (∼2.3-fold after exposure to hypoxia for 48 hours) (Figure 1C) and by immunocytochemistry (Figure 1D).

**Figure 2:**
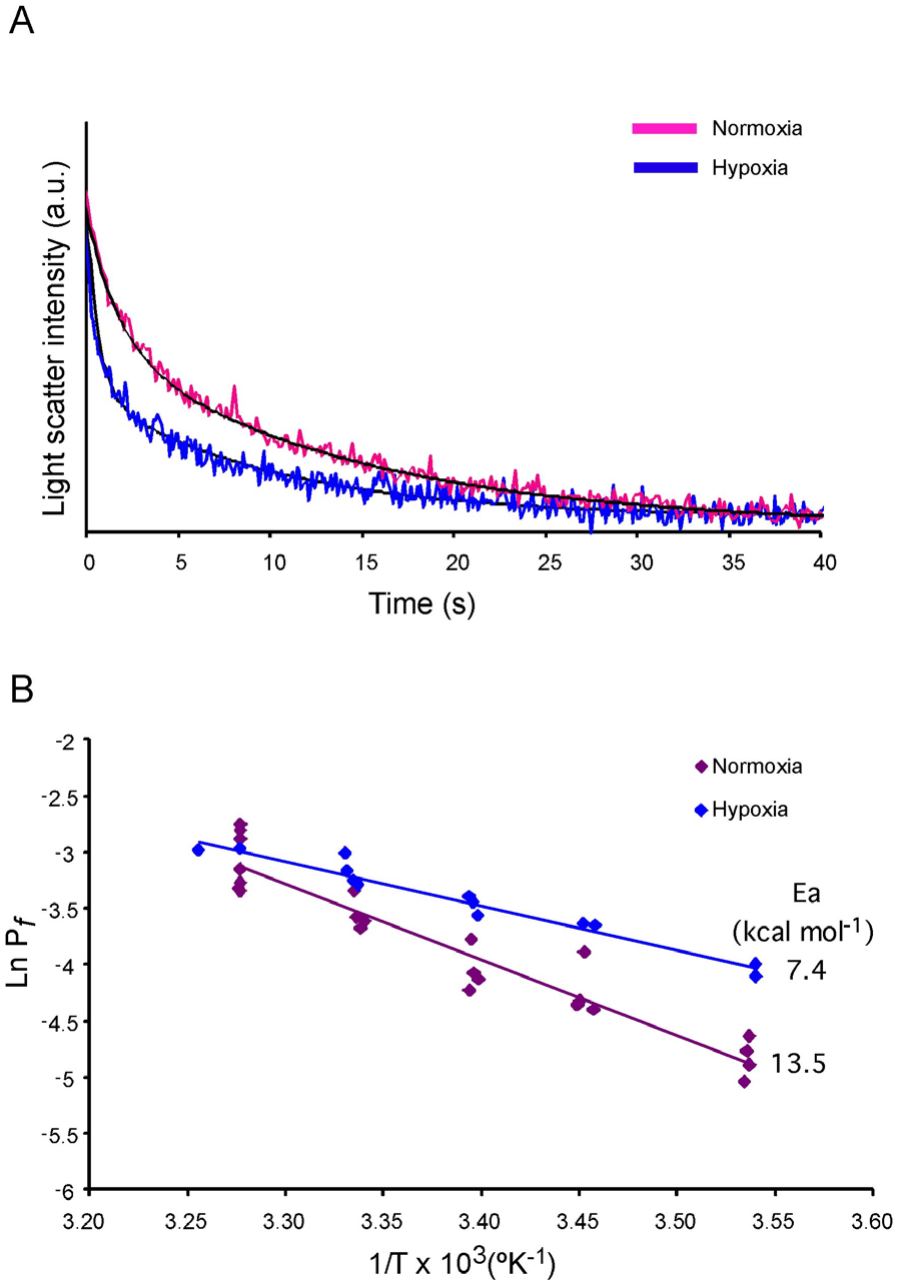
Effect of hypoxia on the water permeability of 9L cells. (A) Normalized scattered light intensity obtained from stopped-flow experiments at 10°C in suspended 9L cells subjected to an osmotic gradient of 150 mOsM/l with mannitol. Cells were pre-exposed in normoxic or 1% O_2_ conditions for 48 h before these experiments. The time course of volume change after the osmotic shock was fitted with a double exponential to calculate P_f_. (B) Temperature dependence of water transport. Typical Arrhenius plots of water efflux from cells maintained in normoxia or treated in hypoxia are shown. From the slope of these plots the activation energy (E_a_) for water transport in 9L cells was calculated. P_f_ and E_a_ values were obtained from at least three independent experiments.

To determine whether the hypoxia-induced Aqp1 protein was functional, plasma membrane water permeability of normoxic and hypoxic 9L cells was assessed using stopped-flow light-scattering. Figure 2A shows a typical recording of normalized scattered light intensity obtained in suspended 9L glioma cells subjected to an osmotic gradient of 150 mOsM/l with mannitol. As described above, the change in light scatter due to cell shrinking was fitted with a double exponential function in order to calculate P_f_. Equilibrium cell volumes, V_o_, measured for both normoxic and hypoxic cells were 1135.1±6.4 and 2227.2±11.4 mm^3^ respectively. A representative Arrhenius plot using P_f_ measurements at five different temperatures from which the E_a_ for water transport was calculated is shown in Figure 2B. P_f_ in hypoxia-treated AQP1 induced cells (19.3±3.4 cm s^−1^) was about 2.5-fold greater than in normoxic cells (7.9±1.3 cm s^−1^) at 10°C, a temperature at which the lipid bilayer contributes less to the bulk permeability thus enabling the functional detection of water channels (Figure 2). The E_a_ value of 13.49 kcal mol^−1^ obtained for normoxic cells is high and similar to the E_a_ for simple lipid membranes where no channels facilitate water transport, whereas a lower E_a_ of 7.44 kcal mol^−1^ obtained for hypoxic cells clearly suggests a larger contribution of molecular channels for water permeation [30]. Specifically, the higher P_f_ and lower E_a_ obtained are compatible with the finding that exposure of 9L glioma cells to hypoxia enhances AQP1 expression with a consequent increase in membrane water permeability.

### Transcriptional Activation of AQP1 Gene by Hypoxia is HIF-1α-Dependent

**Figure 3:**
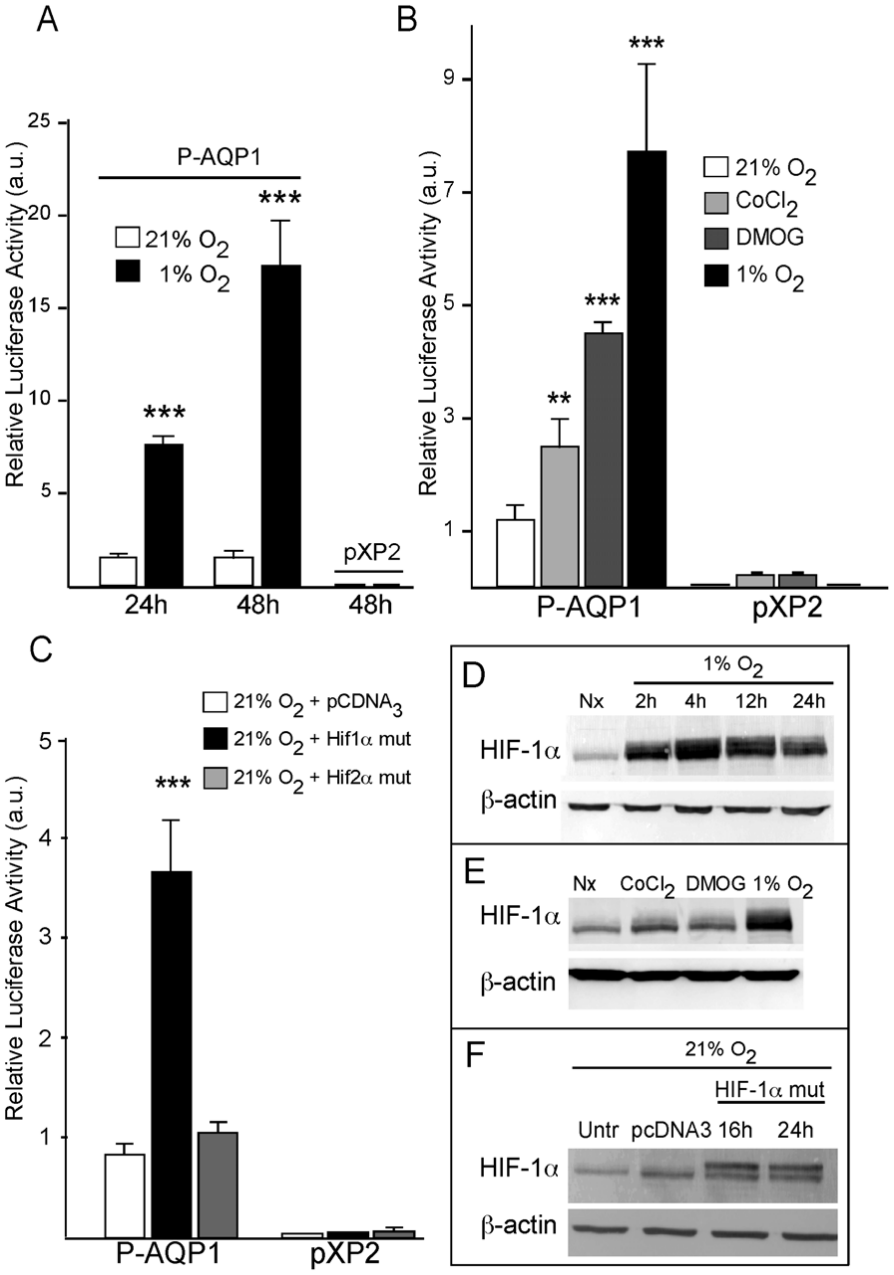
Stabilization of HIF1α- induced transcriptional activity of the Aqp1 promoter. (A) A construct (P-AQP1) in which the reporter firefly luciferase gene was driven by the mouse Aqp1 promoter was transfected into EOMA cells and incubated in normoxia or hypoxia at 1% O_2_, for 24 or 48 h. The values expressed in arbitrary units represent the ratios of firefly luciferase activity normalized by renilla luciferase activity (firefly/renilla). (B) Relative luciferase activity measured in EOMA cells transfected with P-AQP1 and exposed to hypoxia (1% O_2_), normoxia (21% O_2_), or normoxia plus hypoxia mimetics (CoCl_2_ [100 µM] or DMOG [1 mM]) for 24 h. (C) Relative luciferase activity measured from EOMA cells cotransfected with P-AQP1 and mutated forms of HIF1/2α (HIF1/2α mut). Empty pcDNA3 plasmid was used as loading cDNA for control experiments. Cells transfected with the empty vector (pXP2) were used as negative control. Values are expressed in arbitrary units as means ± SEM (n≥3). ** P≤0.01, *** P≤0.001. (D) Western blot time-course analysis of HIF1α protein in EOMA cells kept in normoxia or exposed to hypoxia (1% O_2_) for 2, 4, 12 and 24 h. (E) Analysis by western blot of HIF1α protein in EOMA cells after 4 h with CoCl_2_ (100 µM) or DMOG (1 mM) treatments. (F) Western blot analysis of HIF-1α protein levels in normoxic EOMA cells after 16 and 24 h of transfection with HIF-1α mut plasmid. Non-transfected cells and those transfected with empty pcDNA3 vector were used as controls. β-actin served as protein loading control.

To investigate the molecular bases underlying the hypoxic regulation of Aqp1, we performed bioinformatic analysis of the ∼1.3 Kb promoter region proximal to the initiation codon ATG of Aqp1 (see Supplementary text), looking for the presence of putative DNA binding sites for transcription factors. Several consensus sites for transcription factors involved in regulation by hypoxia of different genes, namely AP1, EGRF, CREB, ETSF, SP1 and NFΚβ [31], were found. More significantly, however, we identified the presence of three “XCGTG” or “HIF binding sites (HBS)” corresponding to the reported consensus binding sequence for HIF (hypoxia-inducible factor), located at positions −1105, −1044 and −157 bp (Supplementary Figure S2),

To analyze Aqp1 regulation by hypoxia, a reporter vector was constructed (P-AQP1) fusing the PCR generated mouse Aqp1 promoter (∼1.3 Kb) to the firefly luciferase gene. Functional response of the Aqp1 promoter was assessed in terms of the luciferase activity observed upon transfection in a murine hemangioendothelioma cell line (EOMA). Luciferase activity derived from Aqp1 promoter activation increased in hypoxia compared to normoxia in a time-dependent manner (Figure 3A), and was higher at 1% than at 3% O_2_ (results not shown). These findings demonstrated a clear response of the Aqp1 promoter to hypoxic conditions and suggested the involvement of HIF in this process. We also analyzed the effect on Aqp1 promoter activity of the hypoxia mimetics CoCl_2_ and DMOG, which inhibit the prolyl hydroxylases and, therefore, prevent HIF hydroxylation and its proteasomal degradation [15], [32]. Luciferase activity of the Aqp1 promoter construct measured after 24 hours of treatment was found to be significantly increased by 100 µM CoCl_2_ (>2-fold) and by 1 mmol/L DMOG (>4-fold), and even more by hypoxia (>7-fold) (Figure 3B). No changes in basal luciferase activity were observed when the promoterless construct (pXP2) was expressed as control. These results strongly indicated that HIF-1α is involved in the hypoxic up-regulation of Aqp1. Promoter-dependent luciferase activity was also evaluated by co-transfecting cells with the mutated forms of Hif-1α and Hif-2α (HIF-1α mut, HIF-2α mut), which are resistant to normoxic prolyl-hydroxylation and subsequent degradation [25], [26]. Transfection of HIF-1α mut, but not HIF-2α mut, produced significant induction (>3-fold) of luciferase activity in normoxic EOMA cells (Figure 3C), thus confirming the direct participation of HIF-1α in the hypoxic up-regulation of Aqp1 promoter.

In parallel with Aqp1 promoter activation, in EOMA cells hypoxia induced a clear stabilization of HIF-1α (Figures 3D–3F). The HIF-1α level reached a peak after 4 hours of hypoxia treatment and gradually decreased over the following 24 hours (Figure 3D). Incubation in CoCl_2_ and DMOG over the same period of time (Figure 3E) also stabilized HIF-1α but to a lesser extent than hypoxia. Moreover, levels of HIF-1α due to co-expression of HIF-1α mut in cells kept in normoxic conditions were clearly higher than those observed in untransfected or pcDNA3 transfected cells (Figure 3F).

### Down-regulation of HIF Reduces Hypoxic Induction of Endogenous AQP1 Expression

**Figure 4:**
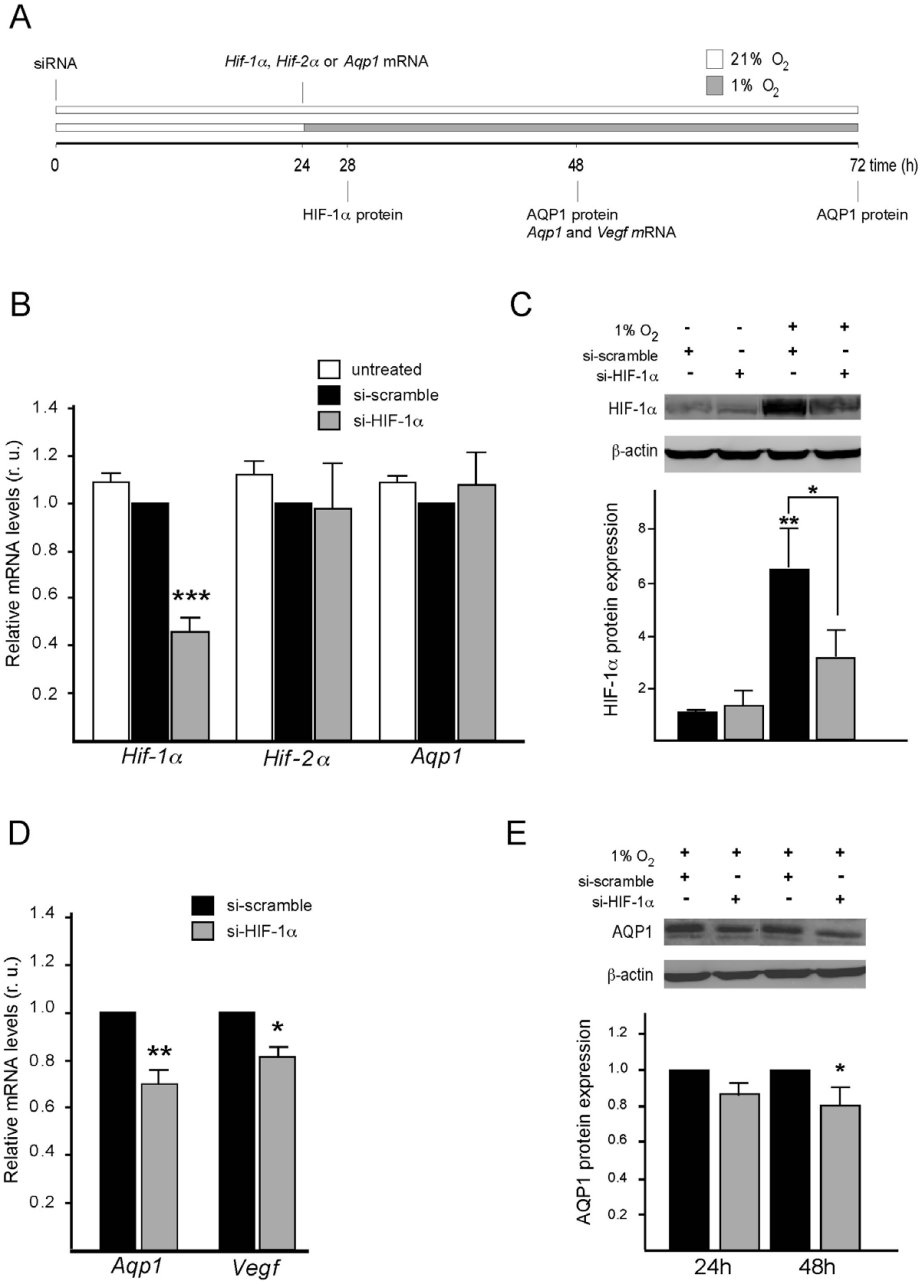
Inhibition by Hif-1α siRNA of Aqp1 mRNA and protein expression. (A) Schematic diagram of the protocol used in the Hif-1α siRNA experiments. Samples were preincubated for 24 h with 50 nmol/L of either Hif-1α siRNA or scramble siRNA. (B) After 24 h of interference, mRNA levels of Hif-1α, Hif-2α or Aqp1 were analyzed by RT-qPCR. (C) Hypoxic conditions (1% O_2_) were applied, and 4 h later, the levels of HIF-1α protein were analyzed by western blot. Levels were quantified from three independent experiments (N = 3). (D), After 48 h (24 h of Hif-1α interference and 24 h of hypoxia) mRNA levels of Aqp1 and Vegf were analyzed by RT-qPCR and normalized by values determined with the scramble siRNA. (E) A representative western blot showing levels of Aqp1 protein in cells treated with Hif-1α siRNA or with scramble siRNA after 48 h in hypoxia. Results of three western blot experiments (N = 3) are summarized here. β-actin was used as protein loading control. Values are presented as means ± SEM (n = 4). * P≤0.01, ** P≤0.001.

To investigate the involvement of HIF-1α in the *in vitro* hypoxia-induced Aqp1 expression in 9L cells, we performed experiments knocking down HIF-1α with specific siRNA. The experimental protocol followed in these experiments is shown in Figure 4A. Cells were co-transfected with 50 nM of specific or scramble siRNA and, after 24 hours of transfection, they were either kept in normoxic or incubated in hypoxic conditions for an additional 48 hours. First, the specificity and efficiency of the interference was confirmed by RT-qPCR analysis of mRNA levels (Figure 4B) and by western blot analysis of HIF-1α stability (Figure 4C). About 60% inhibition of Hif-1α mRNA expression was observed in cells treated with specific siRNA when compared to scramble-treated cells, whereas no effect was detected on Hif-2a or Aqp1 mRNA expression (Figure 4B). Likewise, stability of HIF-1α protein after hypoxic treatment (4 hours at 1% O_2_) was significantly diminished (∼50%) by specific interference compared to levels measured in scramble-treated cells (Figure 4C). We were, therefore, able to conclude that the Hif-1α siRNA used here was specific and effectively reduced expression of this gene. The next step was to analyze the effect that down-regulation of Hif-1α had on the hypoxia-induced overexpression of endogenous Aqp1. In 9L cells treated with Hif-1α siRNA and incubated in hypoxia for 24 hours, levels of Aqp1 mRNA determined by RT-qPCR were 30% lower than those in scramble-transfected cells (Figure 4D). Similarly, levels of Aqp1 protein after 24 and 48 hours of Hif-1α interference were also reduced compared to controls (Figure 4E). These results strongly suggested the involvement of HIF-1α in the mechanism of hypoxia-induced AQP1 expression *in vivo*.

### Mutation and Deletion Analyses of the Aqp1 Promoter

**Figure 5:**
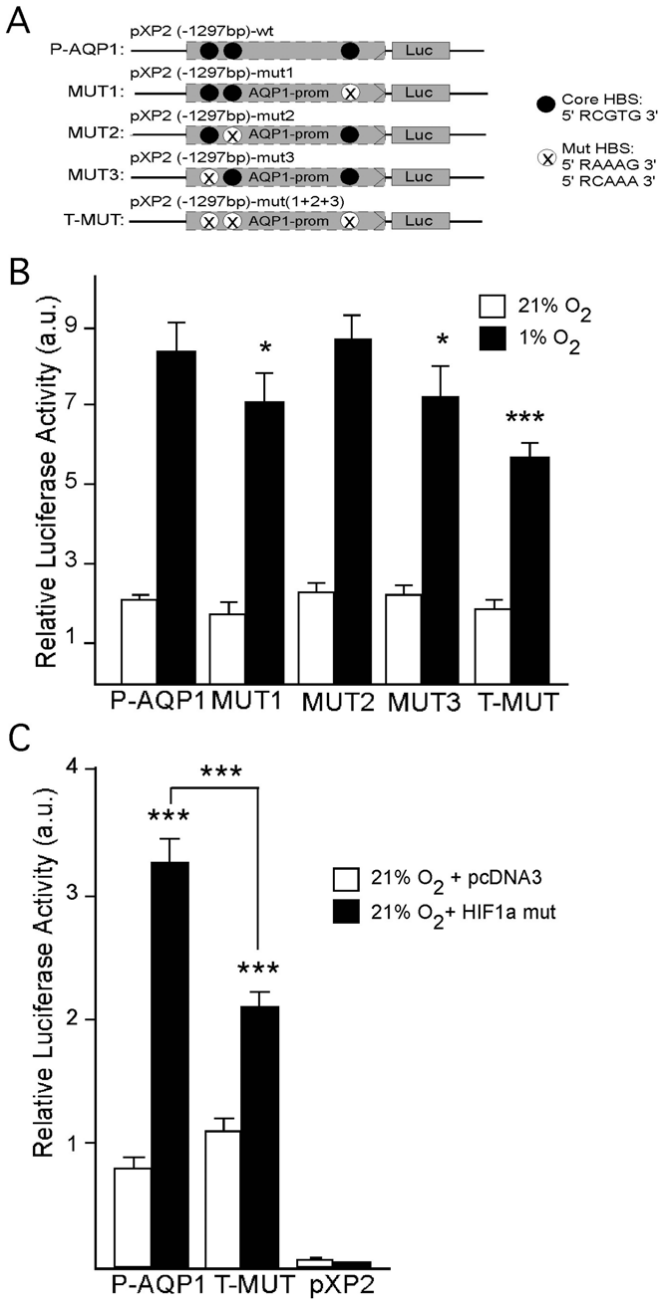
Functional analysis of putative HIF Binding Sites (HBS) in the Aqp1 promoter. (A) The three CGTG sites present in P-AQP1 were individually mutated to AAAG (MUT1, MUT2 and MUT3) by site-directed mutagenesis. The three sites were mutated at once on the triple mutant (T-MUT). (B) Wild type (P-AQP1) or mutated constructs were transfected into EOMA cells and incubated for 24 h either in normoxic (21% O_2_) or at 1% O_2_ conditions before luciferase activity measurements. Values are presented as means ± SEM (N = 4). (C) Relative luciferase activity measured from EOMA cells cotransfected with either P-AQP1 or T-MUT together with HIF-1α mut or pcDNA3 empty plasmid. Cells were maintained under normoxic conditions for 24 h. Values are presented as means ± SEM (N = 5)* P≤0.05, ***P≤0.001.

To determine the actual contribution of HIF-1α to hypoxic activation of the Aqp1 promoter, the three putative HBS identified in the mouse promoter by analysis *in silico* were deleted by site-directed mutagenesis either separately (MUT1, MUT2 and MUT3) or all at the same time (T-MUT) (Figure 5A). Luciferase activity of these constructs in response to hypoxia was measured. Mutations in the first (MUT1) or the third (MUT3) HBS (located at positions −1105/−1109 bp and −157/−161 bp, respectively) resulted in a reduction (∼15%) in luciferase activity in response to hypoxia when compared to the activity shown by the wild type or non-mutated Aqp1 promoter (P-AQP1) (Figure 5B). By contrast, mutation of the HBS located at position −1044/−1048 bp (MUT2) had no effect on the promoter response to hypoxia (Figure 5B). In the triple mutant hypoxia-induced promoter activity diminished by ∼33%, this reduction being equivalent to the sum of the individual effects produced by MUT1 and MUT3 separately (Figure 5B). These results indicated that at least two of the three HBS present in the Aqp1 promoter participate in HIF-mediated regulation of the AQP1 gene.

As an alternative approach to assess whether the HBS mutations explored above did indeed decrease hypoxia induction of the promoter by preventing the binding of HIF-1α and not other transcription factors, we investigated whether the capacity of the triple mutant to respond to HIF-1α mut, rather than hypoxia, was also diminished. Luciferase activity of the two promoters, wild type and triple mutant, was induced in normoxia by co-expression of HIF-1α mut. The level of induction in the triple mutant promoter was only half that observed in the wild type promoter (Figure 5C). These results suggested that HIF-1α binds directly to the HBS on the promoter and that this interaction is prevented in mutated HBS, thus decreasing the transcriptional response to hypoxia.

**Figure 6:**
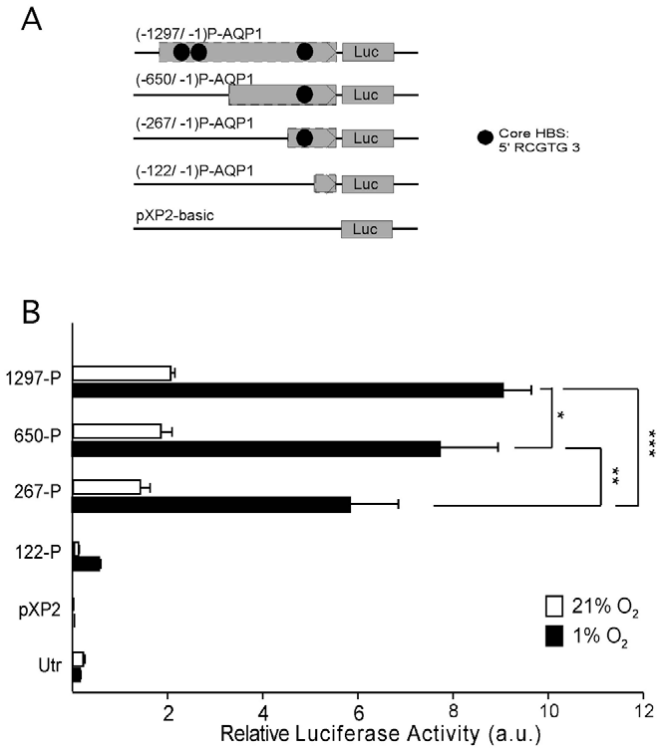
Deletion analysis of the Aqp1 promoter. (A) Schematic diagram of different-size reporter constructs indicating length of the Aqp1 promoter (P-AQP1). (B) Relative luciferase activity measured in EOMA cells transfected with the different constructs and exposed to normoxia or hypoxia (1% O_2_) for 24 h. One-way analysis of variance (ANOVA) followed by Fisher's LSD test was used to evaluate the statistical significance of differences. Bars represent the mean ± SEM for N = 3 independent experiments. *P≤0.05, ** P≤0.01, *** P≤0.001.

On the other hand, using restriction enzymes, we progressively shortened the length of the promoter region, from the longest fragment of 1297 bp to shorter ones of 650, 267 and 122 bp in which only one or none of the HBS remained (Figure 6A). As control, luciferase activity of untransfected cells or transfected with empty pXP2 or pXP2 with short fragments of 93, 63 and 33 bp containing the TATA box region were analyzed. The luciferase activity in response to hypoxia for all deleted forms of the promoter was evaluated and compared to the wild type promoter in simultaneous experiments in which equimolar concentrations of DNA for all constructs were cotransfected. As shown in Figure 6B, the transcriptional response to hypoxia of the promoter 650-P-AQP1 was reduced by 17% with respect to the wild type promoter. This reduction fits very well with the 15% inhibition of luciferase activity observed in MUT3, given that, as demonstrated before, the second HBS (−1044/−1048 bp) mutated in MUT2 does not contribute to the response to hypoxia. Therefore, the first HBS (−1105/−1109) is a functional binding site for HIF-1α. The smaller promoter 267-P-AQP1, although conserving the same HBS (−157/−161) present in 650-P-AQP1, showed a 39% inhibition with respect to the wild type promoter, which suggested the presence of other transcription factors (such as SP-1, EGR-1 or NFΚb) with possible binding sites on the deleted region that also participate in the hypoxic response (Figure 6B). Finally, the construct 122-P-AQP1 in which the remaining HBS was lost, showed not only a dramatic fall in the response to hypoxia but, more importantly, its basal luciferase activity almost disappeared indicating an overall loss of transcriptional activity (Figure 6B). These results support the view that HIF-1α participates in the hypoxic up-regulation of mouse Aqp1, by binding to two of the three HBS (one at −1044/−1048 and the other at −157/−161 bp). However, other hypoxia-inducible transcription factors with binding sequences present on the small remaining fragment of 267-P-AQP1 also seem to participate in the AQP1 gene up-regulation upon exposure to low oxygen conditions.

### Discussion

In the present work, hypoxic induction of AQP1 is shown in several different mouse tissues and cell lines. A functional correlation between hypoxic induction of AQP1 and increases in water permeability is indicated here for the first time, and the involvement of the hypoxia-inducible transcription factor HIF-1α in this phenomenon is analyzed in detail.

### Functional Implications for the Hypoxic Induction of AQP1

Up-regulation of Aqp1 expression by hypoxia was demonstrated in lung and brain tissue of mice exposed to 10% O_2_ for 24 and 48 hours. The increment in Aqp1 mRNA was within the range of the values observed for other hypoxia-inducible genes *in vivo*, although in the lung Aqp1 up-regulation was even higher than that detected for Vegf, used as positive control. Hence, these results suggest an important, previously unreported role for AQP1 in the physiological response to hypoxia of these two organs. Identification of lung cells over-expressing AQP1 under hypoxia was not attempted in the present work. Nonetheless, related research from our laboratory has indicated changes in the expression pattern of AQP1 in lungs of patients with pulmonary diseases that can cause severe hypoxemia, such as cancer and interstitial lung disease [12]. In situ hybridization analysis in brain tissue confirmed the exclusive location of Aqp1 mRNA in choroid plexus epithelium as previously described [33] and, furthermore, demonstrated that over-expression of this gene by hypoxia was predominantly detected in this tissue. It is well known that choroid plexus cells are responsible for the formation of most of the cerebrospinal fluid (CSF) and, further, it has been indicated that the Aqp1 protein participates in CSF secretion, as well as in the modulation of normal values of intracranial pressure [34]–[36].

The results of our stopped-flow light-scattering experiments are consistent with participation of a hypoxic Aqp1 up-regulation in the increase in water permeability observed under such conditions, though further experiments are needed to confirm this. Further, it seems likely that AQP1 is involved in pulmonary or brain edemas produced by hypoxic events, such as those observed in acute mountain sickness [37] or in cerebral ischemia due to vascular injury. Hypoxic induction of AQP1 could also explain the over-expression of this protein observed in several types of human tumors [7]–[12], in which its presence has been always associated with vascular angiogenesis [38] and the rapid water transport necessary for migration and proliferation of new blood vessels [38], [39]. In the case of poorly oxygenated tumors, an increment of Aqp1 expression in the vicinity would explain the typical edematous area surrounding the tumor. Hence, for many of these pathological situations *in vivo* reduction of Aqp1 expression, such as that produced by blocking the transcription factor TTF1 [40] or by inhibiting the JNK signaling pathway [41], might have a useful therapeutic effect.

### HIF-1α-Dependent Hypoxic Up-regulation of the Aqp1 Gene

Promoter analysis of the mouse AQP1 gene confirmed transcriptional up-regulation by hypoxia as indicated by the *in vivo* experiments described before. The promoter activity induced by hypoxia was time- and dose-dependent as occurs with other genes whose expression is modulated by O_2_ concentration [15], [42], [43]. Our in depth analysis of the promoter-luciferase construct has demonstrated that stabilization of the HIF-1α protein, by cobalt chloride and DMOG [15], [32], induces its transcriptional activity. Induction was also observed by transfection with a mutated HIF-1α form resistant to prolyl hydroxylation-dependent degradation. The lack of response to HIF-2α indicates a very selective regulatory mechanism and supports the view that HIF-1α participates in the hypoxic induction of the Aqp1 gene. The results of our siRNA experiments strongly support the idea of HIF-1α having a functional role *in vivo* in Aqp1 up-regulation by hypoxia. Specifically, selective reduction of native Hif-1α diminished hypoxia-dependent induction of endogenous Aqp1 expression at both mRNA and protein levels, confirming direct participation of HIF-1α in that regulatory process.

Moreover, inhibition of promoter activity when the three putative HIF binding sites (CGTG) were mutated provides further evidence for direct binding of HIF-1α but, given the incomplete abrogation of the hypoxia response, also implies the involvement of additional elements in this regulatory mechanism. The fully normal response shown by the MUT2 promoter could well indicate an inactive HBS for Hif-1α binding. It must be noted, however, that as a result of this mutation one specific site for the binding of AP1, a transcription factor also regulated by hypoxia [31], appeared on the promoter offering an alternative explanation for the unexpected result. The smallest hypoxia-responsive unit found on the Aqp1 promoter corresponds to a short region before the first ATG (−267/−1 bp). Our *in silico* analysis of this region revealed, in addition to a HIF-1α binding site (−157/−161 bp), the presence of consensus sequences for binding of hypoxia-responsive transcription factors such as EGR1, SP1, ETS1, AP1, CREB1 and NFκβ. Hence, it is possible that some of these transcription factors participate together with HIF-1α in the hypoxic activation of Aqp1. Indeed, cooperation between HIF-1α and other transcription factors has been established in the hypoxic regulation of several genes [31], [44]–[46]. In line with the lung Aqp1 up-regulation reported here, is well known that AP1, SP1 and CREB are selectively activated in pulmonary hypoxia [17] and could be part of the hypoxic response of AQP1. Furthermore, CREB activation by cAMP and binding of this to HBS [47], [48] in the Aqp1 promoter may constitute an alternative explanation for transcriptional regulation of AQP1 by cAMP [49], [50].

In conclusion, the present work demonstrates that expression of the AQP1 gene is induced in hypoxia by a HIF-1α-dependent mechanism. However, the mutational analysis of the Aqp1 promoter also suggests that besides HIF, other elements contribute to the hypoxic regulation of the AQP1 gene. This observation is important because it could indicate that in genes, such as Aqp1, robustly modulated by hypoxia, multiple transcription factors may participate in this adaptive response. Our study strongly supports the idea that up-regulation of AQP1 by hypoxia may result in increased membrane water permeability. Moreover, it provides a mechanistic framework for understanding the augmented expression of AQP1 associated with tumors and might potentially explain certain cases of edema associated with tissue hypoxia.

## Supporting Information

Figure S1 Hypoxic up-regulation of *Aqp1* in animal tissues and culture cells (A), RT-qPCR analysis showing induction of *Aqp1* and *Vegf* mRNA in lung and brain of mice exposed to hypoxia (10% O_2_ for 24 h, Hy) with respect to normoxia (24 h at 20% O_2_, Nx). (B), RT-qPCR analysis showing induction of Aqp1 in primary culture of rat pulmonary artery smooth muscle cells incubated for 24 h at 3% O_2_. All values were normalized to normoxic levels of mRNA and are presented as means ± SEM (N≥3). *P≤0.05, **P≤0.01, ***P≤0.001. (C), Analysis by *in situ* hybridization of *Aqp1 mRNA* in choroid plexus cells of mice exposed to normoxia or hypoxia (8% O_2_) for 48 h (N = 3).
(TIF)

Figure S2 Schematic diagram of the *Aqp1* gene. (A), *In silico* analysis of murine AQP1 gene indicated that it is constituted by one transcript of 12.12 Kb with four exons separated by three different introns. Three possible HBS (• A/G/TCGTG), the TATA box and the translation start site (A_+1_TG) are indicated over the 1297pb *Aqp1*-promoter. Numbers are relative to the translation start site. (B), Bioinformatic analysis of AQP1 promoter revealed the presence of DNA binding sites for distinct transcription factors that have been implicated in regulation by hypoxia of different genes, such as AP1, CREB, EGRF, ETSF, HIF, NFΚβ, P53 and SP1.
(TIF)

Text S1 Bioinformatic analysis of Aqp1 promoter sequences.
(DOC)

Table S1 Primers to generate Aqp1 promoter mutants.
(DOC)

Table S2 Primers used for qPCR in mouse and rat samples.
(DOC)
