# Supplementary figures and images for: Functional and Transcriptional Induction of Aquaporin-1 Gene by Hypoxia; Analysis of Promoter and Role of Hif-1α
Source: PLoS One. 2011 Dec 7;6(12):e28385. doi: 10.1371/journal.pone.0028385 (PMC3233559; doi:10.1371/journal.pone.0028385)

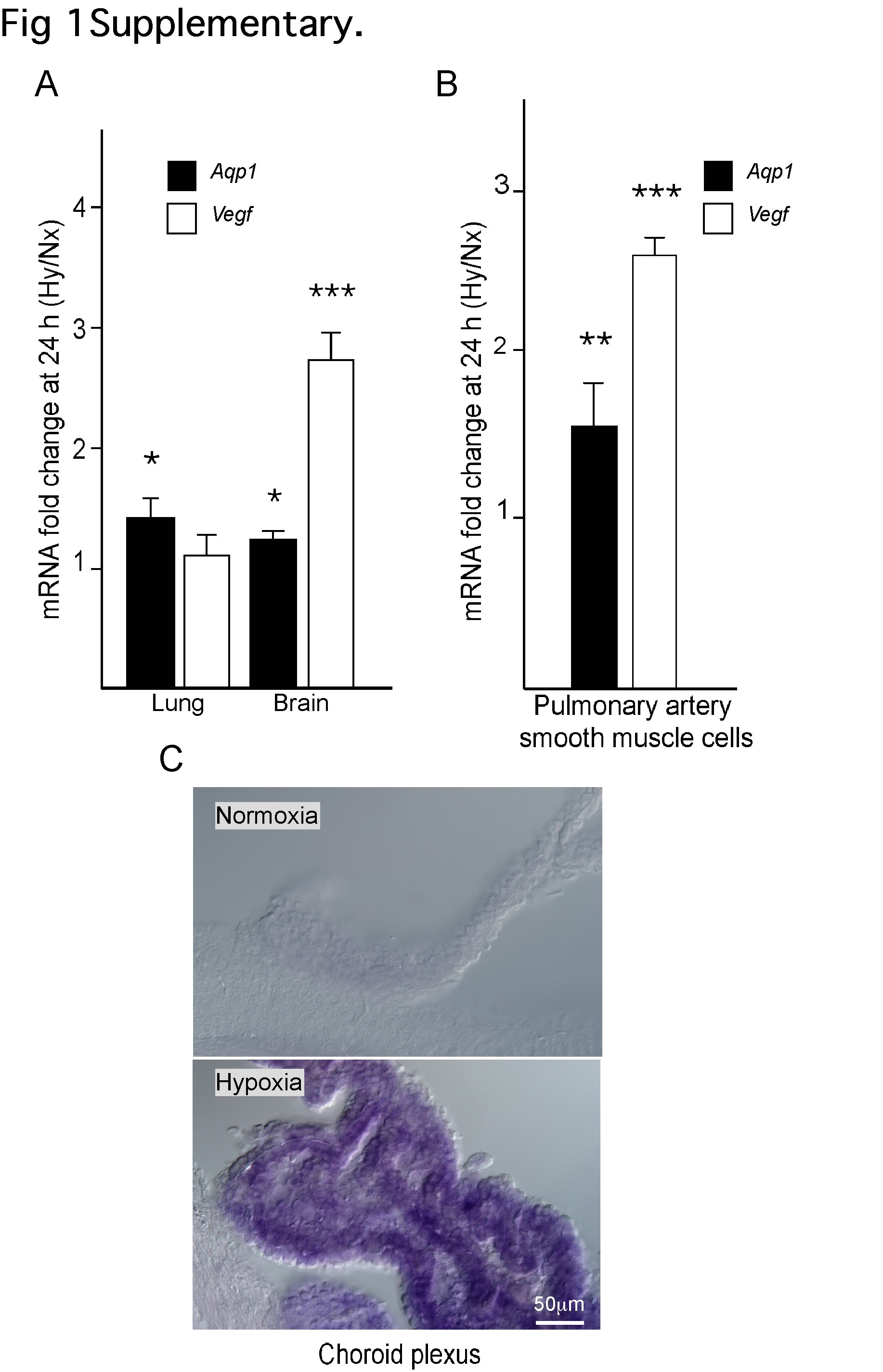

Supplement: Figure S1 — Hypoxic up-regulation of Aqp1 in animal tissues and culture cells (A), RT-qPCR analysis showing induction of Aqp1 and Vegf mRNA in lung and brain of mice exposed to hypoxia (10% O2 for 24 h, Hy) with respect to normoxia (24 h at 20% O2, Nx). (B), RT-qPCR analysis showing induction of Aqp1 in primary culture of rat pulmonary artery smooth muscle cells incubated for 24 h at 3% O2. All values were normalized to normoxic levels of mRNA and are presented as means ± SEM (N≥3). *P≤0.05, **P≤0.01, ***P≤0.001. (C), Analysis by in situ hybridization of Aqp1 mRNA in choroid plexus cells of mice exposed to normoxia or hypoxia (8% O2) for 48 h (N = 3). (TIF) [file pone.0028385.s001.tif]

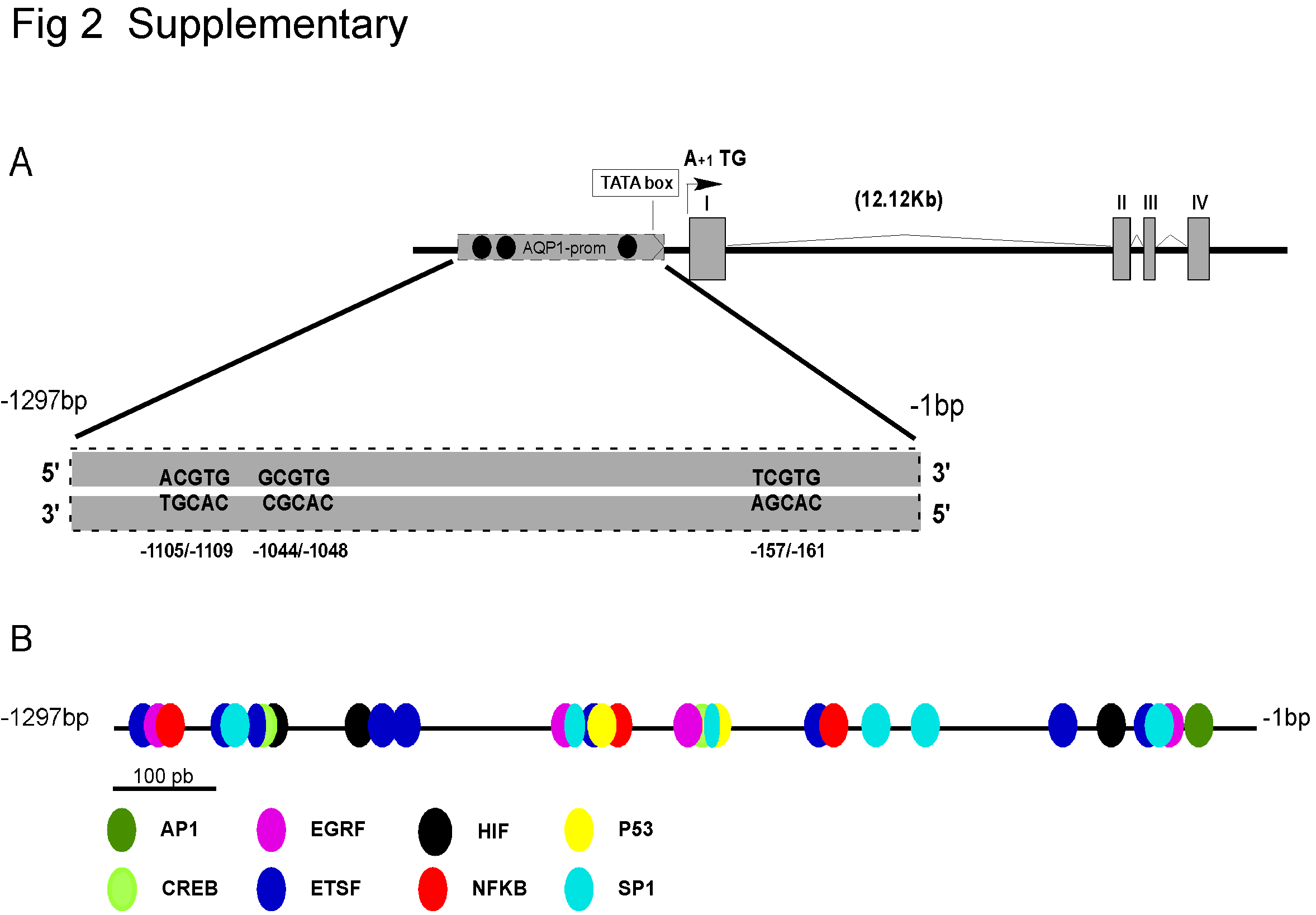

Supplement: Figure S2 — Schematic diagram of the Aqp1 gene. (A), In silico analysis of murine AQP1 gene indicated that it is constituted by one transcript of 12.12 Kb with four exons separated by three different introns. Three possible HBS (• A/G/TCGTG), the TATA box and the translation start site (A+1TG) are indicated over the 1297pb Aqp1-promoter. Numbers are relative to the translation start site. (B), Bioinformatic analysis of AQP1 promoter revealed the presence of DNA binding sites for distinct transcription factors that have been implicated in regulation by hypoxia of different genes, such as AP1, CREB, EGRF, ETSF, HIF, NFΚβ, P53 and SP1. (TIF) [file pone.0028385.s002.tif]
