# Supplementary material for: Functional and Transcriptional Induction of Aquaporin-1 Gene by Hypoxia; Analysis of Promoter and Role of Hif-1α
Source: PLoS One. 2011 Dec 7;6(12):e28385. doi: 10.1371/journal.pone.0028385 (PMC3233559; doi:10.1371/journal.pone.0028385)
Supplement: Text S1 — Bioinformatic analysis of Aqp1 promoter sequences. (DOC) [file pone.0028385.s003.doc]

**Supplementary Text**

Bioinformatic analysis of Aqp1 promoter sequences*.*

*In-silico* analysis of mouse Aqp1 promoter sequence was performed by using the UCSC Genome Browser data base and running specialized bioinformatics programs commonly used to look for putative consensus DNA binding sites for potential regulatory elements present on the promoter region of interest. The programs used were the “CONSITE” (<http://asp.ii.uib.no:8090/cgi-bin/CONSITE/consite/>) [1], the “Transfact” (<http://alggen.lsi.upc.es/cgi-bin/promo_v3/promo/promoinit.cgi?dirDB=TF_8.3>) [2] and “Genomatix” (http://www.genomatix.de/cgi-bin/sessions/login.pl?s=e6df23e0920763addb74aa92c5a69ae1) [3] .

**References**

1. Sandelin A, Wasserman WW and Lenhard B (2004) ConSite: web-based prediction of regulatory elements using cross-species comparison. Nucleic Acids Res, 32: 249-252.

2. Messeguer X, Escudero R, Farre D, Nunez O, Martinez,, J, et al. (2002) PROMO: detection of known transcription regulatory elements using species-tailored searches. Bioinformatics, 18: 333-334.

3. Werner T (2009)The role of transcription factor binding sites in promoters and their *in silico* detection. Bioinformatics for Systems Biology, Stephen Krawetz Ed, Springer Human Press, pp. 339-352.
