# Supplementary material for: Functional and Transcriptional Induction of Aquaporin-1 Gene by Hypoxia; Analysis of Promoter and Role of Hif-1α
Source: PLoS One. 2011 Dec 7;6(12):e28385. doi: 10.1371/journal.pone.0028385 (PMC3233559; doi:10.1371/journal.pone.0028385)
Supplement: Table S1 — Primers to generate Aqp1 promoter mutants. (DOC) [file pone.0028385.s004.doc]

**Supplementary Table S1**

**Table S1. Primers to generate *Aqp1*** promoter mutants

| **MUT1** | F: 5'-CGCAGCGAGGAGGCTC**AAA**TGGTGTGGGGCGGGCC-3' |
| --- | --- |
|  | R:5'-GGCCCGCCCCACACCATTTGAGCCTCCTCGCTGCG-3' |
| **MUT2** | F:5'-AGTGCCCAGTTGAATATTGTATG**AAA**GAGCACATCCATAGCCTG-3' |
|  | R:5'-CAGGCTATGGATGTGCTCTTTCATACAAATATTCAACTGGGCACT-3' |
| **MUT3** | F: 5'-CCGTTTGTCCCTTCCTGTTTCTGAC**AAA**CTGTTTTTTCCTGTG-3' |
|  | R:5'-CACAGGAAAAAACAGTTTGTCAGAAACAGGAAGGGACAAACGG-3' |
