# Supplementary material for: Functional and Transcriptional Induction of Aquaporin-1 Gene by Hypoxia; Analysis of Promoter and Role of Hif-1α
Source: PLoS One. 2011 Dec 7;6(12):e28385. doi: 10.1371/journal.pone.0028385 (PMC3233559; doi:10.1371/journal.pone.0028385)
Supplement: Table S2 — Primers used for qPCR in mouse and rat samples. (DOC) [file pone.0028385.s005.doc]

**Supplementary Table S2**

**Table S2**. Primers used for qPCR in mouse and rat samples

| **Specie/Gene** | **Forward (5´-3´)** | **Reverse (5´-3´)** |
| --- | --- | --- |
| Mouse/*Aqp1* | CATCACCTCCTCCCTAGTC | CATGCGGTCTGTGAAGTCG |
| Rat/ *Aqp1* | GAACTCACTTGGCCGAAATGAC | GCCAGAACGCACAGCACCA |
| Rat/*Hif-1a* | ATCAGCCAGCAAGTCTTCTGA | ATT GACCATATCGCTGTCCACAT |
| Rat/*Hif-2a* | GCAGATGGATAACTTGTACCTGAAAG | CTGACAGAAAGATCATATCACCGTCTT |
| Mouse/ *Cyclophilin* | ATGGCAAATGCTGGACCAAA | TGCCATCCAGCCATTCAGT |
| Rat/ *Cyclophilin* | GCACTGGTGGCAAGTCCAT | GCCAGGACCTGTATGCTTCAG |
| Mouse/Rat/ *Vegf* | CGCAAGAAATCCCGGTTTAA | CAAATGCTTTCTCCGCTC TGA |
| Mouse/Rat/ *18S* | AACGAGACTCTGGCATGCTAACTA | GCCACTTGTCCCTCTAAGAAGTTG |
